# How Plants Control Arsenic Accumulation

**DOI:** 10.1371/journal.pbio.1002008

**Published:** 2014-12-02

**Authors:** Robin Meadows

**Affiliations:** Freelance Science Writer, Fairfield, California, United States of America

In many parts of the world, groundwater contains so much arsenic that it builds up in irrigated crops. Linked to cancer and heart disease, this toxic element is particularly worrisome in rice, which absorbs arsenic more readily than other grains and is a staple for billions of people. Countries with the double whammy of arsenic-laced groundwater and heavy rice consumption include Bangladesh, India, and China.

**Figure 1:**
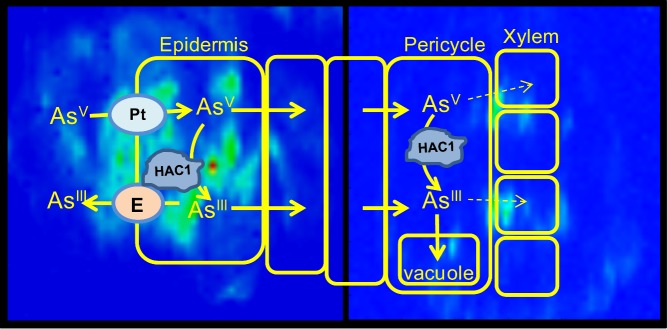
Arsenic is toxic, and its elimination from plants requires it to be converted into arsenite, a form of arsenic that can be released back into the soil from roots. When this fails, arsenic builds up to toxic levels inside the plant.

While plants can detoxify arsenic, we don't know precisely how they do it. In this issue of *PLOS Biology*, the collaborative team of Dai-Yin Chao, Fang-Jie Zhao, and David E. Salt identify an arsenic-reducing enzyme in the plant *Arabidopsis thaliana* and show that this protein is critical to arsenic elimination (Figure 1).

Inorganic arsenic (arsenate) resembles phosphate and, once taken up by roots, likely loads via phosphate transporters into the xylem, which delivers water and nutrients to the shoots. Plants get rid of arsenate by reducing it to arsenite, a form that no longer mimics phosphate and is readily extruded from the roots back into the soil.

To find the enzyme that transforms arsenate into arsenite in plants, the researchers used genome-wide association mapping, which links phenotypes—in this case, arsenic levels in leaves—to genes. They grew 349 types of *A. thaliana* collected from around the world at an environmentally relevant concentration of arsenic, and found that leaf arsenic levels varied more than 20-fold and that this variation was associated with a region of chromosome 2.

Comparison of strains with high and average arsenic levels (Kr-0 and Col-0, respectively) showed that the former has a cytosine at a specific nucleotide in this region, while the latter has a thymine in the same spot. Crossing the two strains showed that arsenic was high in about 25% of the offspring, suggesting that leaf arsenic levels are controlled primarily by a single gene. Named *High Arsenic Content 1* (*HAC1*), this gene has a predicted amino acid domain characteristic of arsenate reductases. To verify that this newly discovered enzyme reduces arsenate to the easily eliminated arsenite, Chao and colleagues expressed *HAC1* in an *Escherichia coli* mutant that lacks its own arsenate reductase. As expected, *HAC1* restored arsenic elimination in this *E. coli* mutant.

In addition, the team found that *HAC1* is expressed in the roots and that root expression rises in *A. thaliana* exposed to arsenate. Moreover, in *A. thaliana* mutants that lack HAC1, arsenic stunted both root and overall plant growth. The latter is important because it shows that HAC1 also keeps arsenic low in shoots, which are often the edible part of a plant. Another important finding is that arsenite extrusion is dramatically reduced in an *HAC1* mutant *A. thaliana* exposed to arsenate, suggesting that this arsenate-reducing enzyme may be coupled with the arsenite efflux transporter.

Besides making a compelling case that HAC1 is part of a major defense against arsenic in plants, Chao and colleagues cleared up a mystery over a previous candidate for this job. Yeast reduces arsenate with an enzyme called ACR2, and initial studies had suggested that plants use a similar enzyme to detoxify arsenic. It turned out, however, that this ACR2-like enzyme reduces arsenate only in vitro and not in living plants. This apparent discrepancy is resolved by the fact that the two plant arsenate reductases (ACR2 and the newly discovered HAC1) share a similar DNA sequence, suggesting that experiments meant to knock out ACR2 actually knocked out HAC1.

To dispel any lingering doubts that *ACR2* may still play a role in plant arsenic detoxification by interacting with *HAC1*, the researchers compared arsenic in an *A. thaliana* mutant that lacks *HAC1* to a double mutant that lacks both *HAC1* and *ACR2*. As expected, arsenic metabolism was similar in the two mutants, confirming that *ACR2* has no impact on arsenic detoxification and elimination in plants.

Using a different method, Eduardo Sánchez-Bermejo and colleagues also recently identified the same gene, which they called *ATQ1*, showing that it encodes an arsenate reductase enzyme involved in plant tolerance to arsenate. However, Chao and colleagues went further by revealing the functional role of HAC1 in arsenic accumulation and arsenate resistance.

The researchers caution that when highly active, this arsenate reducing gene may come at a cost to plants, which could diminish production. That said, their work has tremendous potential to benefit human health. Their key finding—that HAC1 keeps arsenic low in shoots of plants grown at real-world arsenic levels—is a much needed first step toward developing crops that can be grown in high-arsenic regions and still be safe to eat.

**Chao D-Y, Chen Y, Chen J, Shi S, Chen Z, et al. (2014) Genome-wide Association Mapping Identifies a New Arsenate Reductase Enzyme Critical for Limiting Arsenic Accumulation in Plants.** doi:10.1371/journal.pbio.1002009

